# Dr. L. Albert Neisser

**Published:** 1916-09

**Authors:** 


**Dr. L. Albert Neisser,** Prof. of Dermatology at Breslau and known as the discoverer of the diplococcus of gonorrhoea, died in Berlin, July 30.

